# A Novel Mechanism-Equivalence-Based Tweedie Exponential Dispersion Process for Adaptive Degradation Modeling and Life Prediction

**DOI:** 10.3390/s25020347

**Published:** 2025-01-09

**Authors:** Jiayue Wu, Yujie Liu, Han Wang, Xiaobing Ma, Yu Zhao

**Affiliations:** 1 School of Reliability and Systems Engineering, Beihang University, Beijing 100191, China; 2 Reliability and Environmental Engineering Science & Technology Laboratory, Beihang University, Beijing 100191, China

**Keywords:** Tweedie exponential dispersion process, mechanism equivalence, degradation analysis, RUL prediction

## Abstract

Accurately predicting the remaining useful life (RUL) of critical mechanical components is a central challenge in reliability engineering. Stochastic processes, which are capable of modeling uncertainties, are widely used in RUL prediction. However, conventional stochastic process models face two major limitations: (1) the reliance on strict assumptions during model formulation, restricting their applicability to a narrow range of degradation processes, and (2) the inability to account for potential variations in the degradation mechanism during modeling and prediction. To address these issues, we propose a novel mechanism-equivalence-based Tweedie exponential dispersion process (ME-based TEDP) for adaptive degradation modeling and RUL prediction of mechanical components. The proposed model enhances the original Tweedie exponential dispersion process (TEDP) by incorporating degradation mechanism equivalence, effectively capturing the correlation between model parameters. Furthermore, it improves prediction accuracy and interpretability by employing a dynamic testing–modeling–predicting strategy. Application of the ME-based TEDP model to high-speed rail bogie systems demonstrates its effectiveness and superiority over existing approaches. This study advances the theory of degradation modeling and significantly improves the precision of RUL predictions.

## 1. Introduction

### 1.1. Background

Accurate degradation modeling and failure prediction are essential for ensuring the reliability and safety of mechanical systems in high-stakes fields such as aerospace and rail transit [1]. These industries rely on complex mechanical components that operate under time-varying conditions, which makes proactive failure prediction and management critical. Effective failure prediction helps prevent unexpected breakdowns, reduce maintenance costs, and extend the lifetime of critical equipment such as bearings, gears, and treads. A comprehensive understanding of performance degradation can enhance operational safety and efficiency, which are fundamental to success in these challenging sectors.

Despite advancements in state monitoring technologies such as sensors that collect real-time data on parameters such as temperature, vibration, and noise, traditional failure prediction models have notable limitations. Models based on Wiener, gamma, and inverse Gaussian processes (IG process) often exhibit constraints that compromise their accuracy and applicability [2]. These models may fail to fully capture the complexity and variability of mechanical component degradation, resulting in reduced prediction accuracy, poor timeliness, and high false alarm rates. Additionally, many existing approaches do not effectively integrate performance data with the underlying failure mechanisms, leading to suboptimal outcomes in failure prediction and maintenance planning.

To address these challenges, this study introduces the TEDP model as a novel approach for mechanical component degradation modeling and failure prediction. Building on traditional stochastic processes, the TEDP model provides greater flexibility and accuracy in characterizing performance degradation. Compared to other stochastic models, TEDP can capture the stochastic uncertainty of degradation more precisely, thereby improving the failure prediction accuracy [3]. By integrating TEDP with failure mechanism analysis, this study develops an ME-based TEDP model that is better equipped to predict RUL. The ME-based TEDP model provides a flexible tool that better accommodates the stochastic nature of degradation, leading to enhanced prediction precision and more effective maintenance strategies. By enhancing the accuracy and applicability of RUL predictions, the ME-based TEDP model holds significant promise for improving the reliability and safety of mechanical systems in aerospace and rail transit.

### 1.2. Literature Review

Over the past two decades, extensive research has focused on developing degradation models to analyze the performance trends of mechanical components. These models can be broadly categorized into the generalized degradation trajectory model and the stochastic process model [4,5]. The former, which includes exponential, linear, and power-law models, simplifies degradation analysis by illustrating the average degradation paths [6].

Stochastic process models, such as gamma processes, Wiener processes, and inverse Gaussian processes, are used to capture random influences from stress and materials. Wiener processes, characterized by nonmonotonic paths, have been applied in various studies. For instance, Peng and Tseng explored misjudgment effects in linear Wiener processes [6,7], while Liao and Tseng optimized step stress tests using this model [8]. Whitmore adapted Wiener processes with a linear drift term to account for nonlinear degradation, focusing on parameter estimation with measurement errors [9]. Zhang et al. tailored a Wiener process for high-pressure valve degradation, enhancing model accuracy through maximum likelihood estimation [10]. Gamma and inverse Gaussian processes, known for their monotonic trends, are favored where degradation is consistent, as shown by Lawless and Crowder’s work on crack-growth data [11]. To further enhance fault detection, Cheng et al. proposed an improved envelope spectrum method that adaptively selects diagnostic frequency bands, significantly improving the robustness of stochastic models in complex diagnostic tasks [12].

Despite their effectiveness, such models are limited in their ability to handle all scenarios. TEDP integrates gamma, Wiener, and inverse Gaussian processes under varying deviation functions, and has gained attention for its versatility [6]. Zhou and Xu applied TEDP to model physical and chemical property degradation and refined parameter estimation techniques [6]. Duan and Wang extended TEDP to predict residual service life considering random effects and covariates [13], while Luo et al. used TEDP for system reliability assessments in dynamic environments [14]. In dynamic systems, the integration of physical knowledge and machine learning has demonstrated significant promise. For example, Cheng et al. proposed a physics-informed surrogate model that combines Fourier neural operators with residual networks, providing insights into extending TEDP applications to dynamic operational conditions [15]. Mechanical products exhibit multiple failure modes, influenced by stress levels during operation. Ensuring stable failure mechanisms is essential for accurate testing and valid conclusions. Equivalence testing methods evaluate whether these mechanisms remain consistent under stress, typically using parameter equivalence tests based on constant acceleration factors. These tests are based on the principle that if a product’s failure mechanism remains unchanged under varying stress levels, certain mathematical relationships among parameters must remain constant. For example, Sun demonstrated that the coefficient of variation for product life distributions remains stable under unchanged failure mechanisms, regardless of the stress level [16]. Suo et al. extended these principles to Weibull and lognormal distributions, emphasizing the robustness of invariant acceleration principles [17]. Dong et al. derived conditions for parameters in inverse Gaussian processes and used t-statistics to test isotropy in acceleration effects on failure mechanisms [18]. Wang et al. investigated random process degradation models and clarified definitions of acceleration factors and their implications for failure mechanism stability [19]. Collectively, these studies highlight the utility and versatility of parameter equivalence tests based on constant acceleration factors in evaluating changes in failure mechanisms under stress and simplifying testing procedures through focused analysis of life or degradation data from accelerated tests.

Several studies have focused on improving Wiener processes. Wang et al. proposed an improved Wiener process for RUL prediction, where the drift and diffusion parameters are adaptively adjusted based on detection data [20]. Although TEDP models have the potential to unify equivalence conditions in traditional stochastic models, research on their mechanisms and equivalent analysis methods remains limited. Bridging this gap is crucial for advancing failure mechanism analyses within the TEDP framework [3]. To address challenges posed by domain shifts in operational environments, Yan et al. proposed an unsupervised anomaly detection model that combines transformer and dynamic graph convolution, enhancing robustness and offering potential improvements for TEDP models in complex scenarios [21].

In summary, significant progress has been made in developing degradation models and failure mechanism analyses, particularly in applying generalized degradation trajectory models, stochastic processes, and TEDP models. However, challenges persist in addressing complex operational environments and dynamic system behaviors, where domain shifts and varying failure modes undermine the robustness and generalizability of these models. This study aims to bridge these gaps by focusing on failure mechanism equivalence in TEDP models and improving their adaptability through advanced diagnostic methods and machine learning techniques. These efforts aim to expand TEDP’s applicability to diverse operational environments, enabling more precise RUL predictions and improving reliability assessments in mechanical systems.

### 1.3. Overview

This study examines challenges such as unclear failure mechanisms, the limited applicability of existing models, and inadequate prediction accuracy. To address these challenges, an ME-based approach for degradation modeling and RUL prediction has been developed. The proposed approach includes key components such as mechanical inspection, model formulation, parameter estimation, model selection, and RUL prediction. This method was applied to key components of high-speed rail bogie systems, providing theoretical support for more accurate performance degradation modeling and lifetime prediction. The key contributions of this study can be summarized as follows: (1) The ME-based TEDP model, which incorporates mechanism equivalence conditions to better capture parameter correlations and enhance model interpretability, is proposed. It is an improvement over the traditional TEDP model. (2) A dynamic prediction method is developed. As new data become available, the method continuously updates its parameters by analyzing underlying mechanisms, thereby achieving accurate RUL prediction that considers mechanism change in the degradation process.

The rest of the paper is organized as follows: Section 2 discusses the TEDP model and mechanism equivalence conditions. Section 3 outlines the development of the ME-based TEDP model, including special cases and statistical characteristics. Section 4 focuses on statistical methods for parameter estimation and model selection. Section 5 applies the model to predict the RUL in an engineering context.

## 2. TEDP Model and Its Mechanism Equivalence Conditions

### 2.1. Review of TEDP Model

Let $\left\{Y(t),t\geq 0\right\}$ denote the observed degradation path. If the degradation path follows TEDP, Y(t) has the following characteristics:

(1) Y(0)=0;
(2) $\left\{Y(t)|t>0\right\}$ has statistically independent increments;
(3) The probability density function (PDF) of the increment Δy=y(t+Δt)−y(t) is
  (1) $$f(\Delta y;\mu ,\lambda )=c(\Delta y;\lambda ,\Delta t)\cdot \exp\left\{\lambda \left[\Delta y\cdot \omega (\mu )-\Delta t\cdot \kappa (\omega (\mu ))\right]\right\},$$
  where μ is the drift parameter, λ is the dispersion parameter, c(⋅) and κ(⋅) are real functions.

The mean and variance of Y(t) are μt and V(μ)t/λ, where $\mu =\kappa^{\prime }(\omega (\mu ))$, $V(\mu )=\kappa^{\prime \prime }(\omega (\mu ))$, and $\omega^{\prime }(\mu )=1/V(\mu )$. Particularly, $V(\mu )=\mu^{r}$, r∈(−∞,0]∪[1,∞), is defined as the variance function. The Wiener process, the gamma process, and the IG process are special cases of TEDP when r=0,2,3, respectively. Due to the complexity of the TEDP model, the exact expression for the distribution of degradation increment, i.e., f(Δy;μ,λ,r) does not exist. However, using the saddle-point approximation method [22], the approximate expression for f(Δy;μ,λ,r) when λΔt→∞ is given by:

(2) $$f(\Delta y;\mu ,\lambda ,r)=\sqrt{\frac{\lambda }{2\pi \Delta t^{1-r}\Delta y^{r}}}\cdot \exp\left\{-\frac{\lambda \Delta t}{2}d(\Delta y;\Delta t,\mu ,r)\right\}\cdot \left\{1+O(\lambda )\right\},$$

where d(Δy;Δt,μ,r) is the unit deviance function, calculated as:

(3) $$d(\Delta y;\Delta t,\mu ,r)=\left\{\begin{array}{l} {\left(\frac{\Delta y}{\Delta t}-\mu \right)}^{2},r=0\\ 2\left[\frac{\Delta y}{\Delta t}\log\left(\frac{\Delta y}{\mu \Delta t}\right)-\left(\frac{\Delta y}{\Delta t}-\mu \right)\right],r=1\\ 2\left[\log\left(\frac{\mu \Delta t}{\Delta y}\right)+\frac{\Delta y}{\mu \Delta t}-1\right],r=2\\ 2\left\{\frac{\left[\max{\left(\Delta y/\Delta t,0\right)}^{2-r}\right]}{\left(1-r)(2-r\right)}-\frac{\mu^{1-r}\Delta y}{(1-r)\Delta t}+\frac{\mu^{2-r}}{2-r}\right\},r\neq 0,1,2 \end{array}\right..$$

### 2.2. Mechanism Equivalence Conditions for TEDP Model

The lifetime of a product is defined as the first passage time when its degradation path crosses a pre-specified threshold ω, i.e., $T=\inf\left\{t,Y(t)\geq \omega \right\}$. The cumulative distribution function (CDF) and PDF of T can be approximated by the Birnbaum–Saunders distribution (B-S distribution) as:

(4) $$F_{T}(t|\theta )≅\Phi \left[\sqrt{\frac{\lambda }{\mu^{r}}}\left(\mu \sqrt{t}-\frac{\omega }{\sqrt{t}}\right)\right]$$

and

(5) $$f_{T}(t|\theta )=\frac{\mu t+\omega }{2t}\cdot \sqrt{\frac{\lambda }{2\pi \mu^{r}t}}\cdot \exp\left[-\frac{\lambda }{2\mu^{r}}{\left(\mu \sqrt{t}-\frac{\omega }{\sqrt{t}}\right)}^{2}\right]$$

respectively, where θ=(μ,λ,r), Φ(⋅) is the CDF of the standard normal distribution [23].

Assuming that the distribution functions for the first passage time of the product at two different stress levels are equal, $F_{T}(t_{i}|\theta_{i})=F_{T}(K_{ij}t_{i}|\theta_{j})$, the ratio of these two first passage times $K_{ij}$—defined as the acceleration degradation factor of stress level $s_{i}$ for stress level $s_{j}$—should satisfy the following formula:

(6) $$f_{T}(t_{i}|\theta_{i})=K_{ij}f_{T}(K_{ij}t_{i}|\theta_{j})$$

where $f_{T}(t_{i}|\theta_{i})$ and $f_{T}(K_{ij}t_{i}|\theta_{j})$ represent the PDF at $s_{i}$ and $s_{j}$, respectively.

According to the acceleration factor invariant principle, the value of the acceleration degradation factor should depend solely on the stress level [24]. If the failure mechanism of the product remains unchanged under different stresses, all coefficients containing $t_{i}$ in $f_{T}(t_{i}|\theta_{i})/f_{T}(K_{ij}t_{i}|\theta_{j})$ must equal zero to ensure that $K_{ij}$ is solely determined by the stress level. Based on this, the quantitative relationship among performance degradation model parameters at various stress levels can be derived.

According to Equation (5), the PDF of T under the TEDP can be approximated by the B-S distribution. For TEDP, replacing the PDF of life T under two different stress levels with the acceleration factor invariant principal yields:

(7) $$\begin{array}{l} K_{ij}=\frac{f_{T}(t_{i}|\theta_{i})}{f_{T}(K_{ij}t_{i}|\theta_{j})}=\frac{\frac{\mu_{i}t_{i}+\omega }{2t_{i}}\sqrt{\frac{\lambda_{i}}{2\pi \mu_{i}^{r_{i}}t_{i}}}\exp\left[-\frac{\lambda_{i}}{2\mu_{i}^{r_{i}}}{\left(\mu_{i}\sqrt{t_{i}}-\frac{\omega }{\sqrt{t_{i}}}\right)}^{2}\right]}{\frac{\mu_{j}K_{ij}t_{i}+\omega }{2K_{ij}t_{i}}\sqrt{\frac{\lambda_{j}}{2\pi \mu_{j}^{r_{j}}K_{ij}t_{i}}}\exp\left[-\frac{\lambda_{j}}{2\mu_{j}^{r_{j}}}{\left(\mu_{j}\sqrt{K_{ij}t_{i}}-\frac{\omega }{\sqrt{K_{ij}t_{i}}}\right)}^{2}\right]}\\ ={K^{3/2}}_{ij}\cdot \frac{\mu_{i}t_{i}+\omega }{\mu_{j}K_{ij}t_{i}+\omega }\cdot \sqrt{\frac{\lambda_{i}\mu_{j}^{r_{j}}}{\lambda_{j}\mu_{i}^{r_{i}}}}\cdot \exp\left[\frac{\lambda_{j}}{2\mu_{j}^{r_{j}}}{\left(\mu_{j}\sqrt{K_{ij}t_{i}}-\frac{\omega }{\sqrt{K_{ij}t_{i}}}\right)}^{2}-\frac{\lambda_{i}}{2\mu_{i}^{r_{i}}}{\left(\mu_{i}\sqrt{t_{i}}-\frac{\omega }{\sqrt{t_{i}}}\right)}^{2}\right]\\ ={K^{3/2}}_{ij}\cdot \frac{\mu_{i}t_{i}+\omega }{\mu_{j}K_{ij}t_{i}+\omega }\cdot \sqrt{\frac{\lambda_{i}\mu_{j}^{r_{j}}}{\lambda_{j}\mu_{i}^{r_{i}}}}\cdot \exp\left[\frac{\lambda_{j}}{2\mu_{j}^{r_{j}}}\left(\mu_{j}^{2}K_{ij}t_{i}+\frac{\omega^{2}}{K_{ij}t_{i}}-2\mu_{j}\omega \right)-\frac{\lambda_{i}}{2\mu_{i}^{r_{i}}}\left(\mu_{i}^{2}t_{i}+\frac{\omega^{2}}{t_{i}}-2\mu_{i}\omega \right)\right]\\ ={K^{3/2}}_{ij}\cdot \frac{\mu_{i}t_{i}+\omega }{\mu_{j}K_{ij}t_{i}+\omega }\cdot \sqrt{\frac{\lambda_{i}\mu_{j}^{r_{j}}}{\lambda_{j}\mu_{i}^{r_{i}}}}\cdot \exp\left[\left(\frac{\lambda_{j}K_{ij}}{\mu_{j}^{r_{j}-2}}-\frac{\lambda_{i}}{\mu_{i}^{r_{i}-2}}\right)\frac{t_{i}}{2}+\left(\frac{\lambda_{j}}{\mu_{j}^{r_{j}}K_{ij}}-\frac{\lambda_{i}}{\mu_{i}^{r_{i}}}\right)\frac{\omega^{2}}{2t_{i}}-\left(\frac{\lambda_{j}}{\mu_{j}^{r_{j}-1}}-\frac{\lambda_{i}}{\mu_{i}^{r_{i}-1}}\right)\omega \right] \end{array}$$

The above formula must hold at all times. Therefore, all terms including $t_{i}$ must either be eliminated or have zero coefficients, resulting in:

(8) $$\frac{\lambda_{j}K_{ij}}{\mu_{j}^{r_{j}-2}}-\frac{\lambda_{i}}{\mu_{i}^{r_{i}-2}}=0,\frac{\lambda_{j}}{\mu_{j}^{r_{j}}K_{ij}}-\frac{\lambda_{i}}{\mu_{i}^{r_{i}}}=0.$$

This formula is equivalent to:

(9) $$K_{ij}=\frac{u_{i}}{u_{j}},\frac{\mu_{i}^{r_{i}-1}}{\mu_{j}^{r_{j}-1}}=\frac{\lambda_{i}}{\lambda_{j}}.$$

For a specific accelerated degradation test, if TEDP is used to characterize the degradation process and the degradation mechanism remains unchanged, the accelerated degradation factor should satisfy the above relationship.

## 3. The ME-Based TEDP Model

### 3.1. ME-Based TEDP Model Construction

By slightly transforming Equation (9), the definition of the mechanical equivalence factor (MEF) is obtained:

(10) $$m=\frac{\lambda }{\mu^{r-1}}.$$

According to the definition of MEF, if the degradation mechanism remains unchanged, it should theoretically be a constant during the degradation process. Based on the failure mechanism equivalence principle, the TEDP is analyzed to derive the constraint relationship among its parameters. According to the MEF, the expression of the dispersion parameter λ can be derived, which is represented as Equation (11) in terms of m, μ, r:

(11) $$\lambda =m\mu^{r-1}.$$

Among them, m is a constant, and λ is only a function of μ and r.

Traditional stochastic processes often overlook the failure mechanism equivalence principle. Incorporating the dispersion parameter expression derived from the necessary condition for mechanism equivalence into the TEDP model, enables the construction of an improved ME-based TEDP model, denoted as $Μ\left(m,\mu ,r\right)$. The PDF of the degradation increment for the ME-based TEDP model is:

(12) $$f\left(\Delta y;\mu ,r,m\right)=\sqrt{\frac{m{\left(\mu \Delta t\right)}^{r-1}}{2\pi \Delta y^{r}}}\cdot \exp\left\{-\frac{m\mu^{r-1}\Delta t}{2}d\left(\Delta y;\Delta t,\mu ,r\right)\right\}\cdot \left\{1+O(m\mu^{r-1})\right\}.$$

Among them, d(Δy;Δt,μ,r) is the unit deviation function, and the calculation formula remains unchanged. The three commonly used stochastic processes, the Wiener process, the gamma process, and the IG process, are special cases of the TEDP model at r=0,2,3, respectively.

### 3.2. Special Cases of the ME-Based TEDP Model

After constructing the ME-based TEDP model, traditional stochastic processes also undergo corresponding changes. The relationship between three commonly used stochastic processes and the ME-based TEDP model is shown in Table 1.

- Special case 1. ME-based Wiener process

For the Wiener process, r=0, thus $m=\lambda \mu =\eta /\sigma^{2}$ can be derived. Consequently, the ME-based Wiener process model is expressed as:

(13) $$Y(t)=m\sigma^{2}t+\sigma B(t).$$

Its degradation increment PDF is expressed as:

(14) $$f^{Wiener}(\Delta y)=\sqrt{\frac{1}{2\pi \sigma^{2}\Delta t}}\cdot \exp\left\{-\frac{\Delta t}{2\sigma^{2}}\cdot {\left(\frac{\Delta y}{\Delta t}-m\sigma^{2}\right)}^{2}\right\}.$$

- Special case 2. ME-based gamma process

For the gamma process, r=2, thus $m=\lambda /\mu =\beta_{2}$ can be derived. Consequently, the ME-based gamma process model is expressed as:

(15) $$Y(t)\sim \Gamma (t;\beta_{1},m).$$

Its degradation increment PDF is expressed as:

(16) $$f^{Gamma}(\Delta y)=\sqrt{\frac{\beta_{1}\Delta t}{2\pi \Delta y^{2}}}\cdot \exp\left\{-\beta_{1}\Delta t\left[\log\left(\frac{\beta_{1}\Delta t}{m\Delta y}\right)+\frac{m\Delta y}{\beta_{1}\Delta t}-1\right]\right\}.$$

- Special case 3. ME-based IG process

For the IG process, r=3, thus $m=\lambda /\mu^{2}=\delta_{2}/\delta_{1}^{2}$ can be derived. Consequently, the ME-based IG process model is expressed as:

(17) $$Y(t)\sim IG(t;\delta_{1},m\delta_{1}^{2}).$$

Its degradation increment PDF is expressed as:

(18) $$f^{IG}(\Delta y)=\sqrt{\frac{m{\left(\delta_{1}\Delta t\right)}^{2}}{2\pi \Delta y^{3}}}\cdot \exp\left\{-m\delta_{1}^{2}\Delta t\left[\frac{1}{2\max(\Delta y/\Delta t,0)}+\frac{\Delta y}{2\delta_{1}^{2}\Delta t}-\frac{1}{\delta_{1}}\right]\right\}.$$

Wang et al. previously proposed improvements to the Wiener process [20], which represent a special case of the enhanced TEDP model introduced in this paper.

## 4. Adaptive Degradation Modeling and RUL Prediction Using the Proposed Model

**Figure 1:**
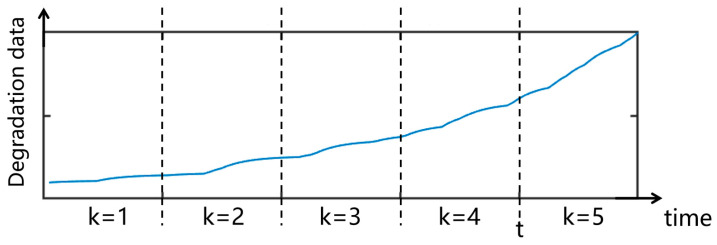
Schematic diagram of the mechanism testing process.

Assuming that the collected data are as shown in Figure 1, a specific point in time t is defined. For new data collected after time t, a comparison is made with the data collected before t to determine whether the degradation mechanisms are equivalent. If the mechanisms are equivalent, the new data can be combined with the old data. If they differ, careful consideration must be given to judge whether the new data should be used for RUL prediction. Statistically, if degradation mechanisms remain unchanged across different phases, the MEF should exhibit statistical consistency; significant differences in MEF indicate changes in the underlying mechanisms. This section establishes a method for identifying degradation mechanism equivalence and utilizing the tested data to perform RUL prediction.

Assuming that a total of n products’ degradation data are collected and divided into k degradation phases based on time series, $c_{i}$ measurements are taken in each stress phase, i.e., $h=1,2,\cdots ,c_{i}$. Represent the degradation phases as i, i=1,2,⋯,k, and the unit number as j, $j=1,2,\cdots ,n_{i}$. For convenience, the historical monitoring time and corresponding degradation path are denoted as $T_{0\to k}=\left\{t_{0},t_{1},\cdots ,t_{k}\right\}$ and $Y_{0\to k}=\left\{y_{0},y_{1},\cdots ,y_{k}\right\}$ respectively, where $t_{i}$ represents the start or end time of each degradation phase, $y_{i}=Y(t_{i})$.

### 4.1. Parameter Estimation

Previous studies have proposed various parameter estimation methods for stochastic process models, including maximum likelihood estimation, least squares method, expectation-maximization algorithm, and Monte Carlo method. Considering the large sample size and the complexity of the model, this study adopts the classical maximum likelihood estimation method. The unknown parameters to be estimated are μ, r and m.

Let $\Delta t_{i}=t_{i}-t_{i-1}$ and $\Delta y_{i}=y_{i}-y_{i-1}$, then the log-likelihood function of the product degradation path is:

$$\begin{array}{l} \ln L(Y_{0\to i},\Theta )=\sum_{h=1}^{c_{i}}Inf(\Delta y_{h};\Theta )\\ =\sum_{h=1}^{c_{i}}\left\{\frac{1}{2}\left[\ln m+(r-1)\ln(\mu \Delta t_{h})-\ln(2\pi )-r\ln\Delta y_{h}\right]-\frac{m\mu^{r-1}\Delta t_{h}}{2}d(\Delta y_{h};\Delta t_{h},\mu ,r)\right\}\\ =\frac{c_{i}}{2}\ln\frac{m}{2\pi }+\frac{(r-2)c_{i}}{2}\ln\mu +\sum_{h=1}^{c_{i}}\left(\frac{r-1}{2}\ln\Delta t_{h}-\frac{r}{2}\ln\Delta y_{h}\right)-\frac{m\mu^{r-1}}{2}\sum_{h=1}^{c_{i}}\left(\Delta t_{h}d(\Delta y_{h};\Delta t_{h},\mu ,r)\right) \end{array}$$

Take the first-order partial derivative of $\ln L(Y_{0\to k},\Theta )$ with respect to μ and m, respectively, then we have:

(19) $$\hat{\mu }=\frac{\sum_{h=1}^{c_{i}}\Delta y_{c_{i}}}{\sum_{h=1}^{c_{i}}\Delta t_{c_{i}}}.$$

(20) $$\hat{m}=\frac{c_{i}}{\sum_{h=1}^{c_{i}}\Delta t_{c_{i}}d(\Delta y_{c_{i}};\Delta t_{c_{i}},\hat{\mu }){\hat{\mu }}^{r-1}}.$$

According to the above formulas, $\hat{\mu }$ can be directly calculated, but $\hat{m}$ is a function of r. Substituting the expressions for $\hat{\mu }$ and $\hat{m}$ into the likelihood function, the fmincon function in MATLAB (R2020a) is used to find the minimum point of the negative likelihood function and solve for $\hat{r}$. Finally, substitute $\hat{r}$ back to the $\hat{m}$ expression to calculate the estimated values of all parameters.

### 4.2. Mechanism Test for Different Degradation Phases

In Section 3.1, the relationship between MEF and parameters μ, λ, and r in the TEDP model was analyzed. According to the definition of MEF, this factor determines whether the failure mechanism of products changes across different degradation phases. In practice, the MEF derived from engineering data is influenced by random factors and thus behaves as a random variable. Based on the statistical analysis of MEF equivalence across different degradation stages, this paper proposes a method for mechanism equivalence testing, which consists of the following three main steps.

(1) Construction of MEF Samples

The parameters of each phase and product are estimated separately, and k MEF sample vectors are constructed as follows, where ${\hat{M}}_{ij}$ represents the MEF of the i-th degradation phase estimated from the j-th unit.

(21) $${\hat{M}}_{i}=\left({\hat{M}}_{i1},{\hat{M}}_{i2},\cdots ,{\hat{M}}_{in_{i}}\right)$$

Denote ${\hat{M}}_{1},\cdots ,{\hat{M}}_{i},\cdots ,{\hat{M}}_{k}$ as the hypothesis testing sample. Since the products in each degradation phase belong to the same batch, the elements in each MEF vector are assumed to originate from the same distribution group. Furthermore, assuming these elements are independent, no correlation exists between the distribution populations that each MEF follows. This enables the identification of significant differences between populations with different distributions using mean and variance tests.

(2) Normality Test of MEF Samples

Various methods exist for testing whether a sample follows a normal distribution. In this study, due to cost constraints, the number of elements in each degradation phase is small. Therefore, the Shapiro–Wilk (SW) test, which performs well in small sample sizes, is considered. The SW test in SPSS (26) software verifies whether the ${\hat{M}}_{i1},{\hat{M}}_{i2},\cdots ,{\hat{M}}_{in_{i}}$ data within a given degradation phase follow a normal distribution.

At a significance level α, the rejection region of the SW test is defined as follows:

(22) $$\Omega_{SW}=\left\{SW_{i}<SW_{i,\alpha }\right\}.$$

where $SW_{i,\alpha }$ denotes the α-quantile of the normal distribution.

(3) Mechanism Equivalence Testing of MEF Samples

If the SW test is accepted in the second step, ${\hat{M}}_{i1},{\hat{M}}_{i2},\cdots ,{\hat{M}}_{in_{i}}$ is considered to follow a normal distribution. Remove degradation phases that fail the SW test, then perform mechanism equivalence testing on the remaining samples. In the i-th degradation phase with a normally distributed population, the following formula is used to obtain unbiased estimates of the mean and variance, where $i=1,2,\cdots ,k^{\prime }$.

(23) $${\bar{p}}_{i}=\frac{1}{n_{i}}\sum_{j=1}^{n_{i}}{\hat{M}}_{ij}$$

(24) $${\bar{q}}_{i}^{2}=\frac{1}{n_{i}-1}\sum_{j=1}^{n_{i}}{\left({\hat{M}}_{ij}-p_{i}\right)}^{2}$$

Furthermore, test whether the distributions of MEF samples across different degradation phases are equivalent. First, the Bartlett test [25] is used to assess variance consistency. The Bartlett statistic is applicable for testing consistency in populations with different normal distributions. The null hypothesis of equal variance is expressed as:

$$H_{0}:q_{1}^{2}=q_{2}^{2}=\cdots =q_{k}^{2}.$$

The Bartlett statistic, denoted as B, is:

(25) $$B=\frac{\nu \ln({\bar{q}}^{2})-\sum_{i=1}^{k}\nu_{i}\ln({\bar{q}}_{i}^{2})}{1+\frac{1}{3(k-1)}\left(\sum_{i=1}^{k}\frac{1}{\nu_{i}}-\frac{1}{\nu }\right)}$$

Among them, ${\bar{q}}^{2}=\left[\sum_{i=1}^{k}\nu_{i}{\bar{q}}_{i}^{2}\right]/\nu$, $\nu_{i}=n_{i}-1$, $\nu =\sum_{i=1}^{k}\nu_{i}$.

At a significance level α, the rejection region is:

(26) $$\Omega_{Bart}=\left\{Β\geq \chi_{\alpha }^{2}(k-1)\right\}$$

Compared to other hypothesis testing methods, such as the F-test [26], the Bartlett test offers higher efficiency [25]. However, if the null hypothesis is rejected, the Bartlett test does not identify specific degradation stages. In such cases, F-tests can be conducted between adjacent degradation stages to pinpoint the exact stage of mechanism change.

Subsequently, degradation phases with inconsistent variances compared to other stages are excluded, and mean equivalence tests are performed on the remaining phases. ANOVA is used to test the means of the samples within each degradation phase. Analysis of variance (ANOVA) is a statistical method for determining significant differences in the means of three or more groups. This method was developed in the 1920s by British scientist Sir Ronald Aylmer Fisher, who analyzed the effects of fertilizers on crop production [27]. ANOVA has the advantage of pinpointing specific stages where the degradation mechanism changes, even if the null hypothesis is rejected.

Finally, data corresponding to the degradation phases with statistically equivalent MEFs are selected for RUL prediction. In these phases, the degradation mechanism is considered stable, enabling data combination without compromising RUL prediction accuracy.

### 4.3. RUL Prediction

Using the degradation monitoring data, a distribution function for the product’s RUL is generated, and a method for residual life prediction is developed. Based on the assumption of equivalent mechanisms and the definition of TEDP, the conditions for failure mechanism equivalence are derived and a residual lifetime prediction equation that incorporates mechanism equivalence is constructed.

The results of mechanism equivalence testing identify the degradation stages where the product’s failure mechanism remains unchanged. These phases are denoted as $s_{1}<s_{2}<\cdots <s_{k^{\prime }}$, where $s_{k^{\prime }}$ represents the last degradation stage with the same mechanism, $k^{\prime }\leq k$. Additionally, the degradation data corresponding to the equivalent failure mechanism are designated as effective degradation data. This effective data will be used in subsequent lifetime prediction processes.

Denote the unknown parameters as $\Theta =\left(\mu_{1},\cdots ,\mu_{k^{\prime }},\lambda_{1},\cdots ,\lambda_{k^{\prime }},M_{1},\cdots ,M_{k^{\prime }},r_{1},\cdots ,r_{k^{\prime }}\right)$, and based on the selected effective degradation data, the complete logarithmic likelihood function can be expressed as:

(27) $$\begin{array}{l} \ln L(\Upsilon ,\Theta )=\sum_{i=1}^{k^{\prime }}\sum_{j=1}^{n_{i}}\sum_{h=1}^{c_{i}}Inf(\Delta y_{ijh};\Theta )\\ =\sum_{i=1}^{k^{\prime }}\left(\frac{n_{i}c_{i}}{2}\ln\frac{\lambda_{i}}{2\pi }\right)-\sum_{i=1}^{k^{\prime }}\sum_{j=1}^{n_{i}}\sum_{h=1}^{c_{i}}\left(\frac{1-r}{2}\ln\Delta t_{ijh}+\frac{r}{2}\ln\Delta y_{ijh}\right)-\sum_{i=1}^{k^{\prime }}\sum_{j=1}^{n_{i}}\sum_{h=1}^{c_{i}}\left(\frac{\lambda_{i}\Delta t_{ijh}}{2}d(\Delta y_{ijh};\Delta t_{ijh},\mu_{i})\right) \end{array}$$

Among them, $\Upsilon =\left\{y_{ijh}|i=1,2,\cdots ,k^{\prime };j=1,2,\cdots ,n_{i},h=1,2,\cdots ,c_{i}\right\}$, $y_{ijh}$ is degradation observed values for the j-th unit under the i-th stress level measured at $t_{ijh}$, $c_{i}$ is the number of measurements of each unit in each degradation phase, $\Delta t_{ijh}=t_{ijh}-t_{ij(h-1)}$, $\Delta y_{ijh}=y_{ijh}-y_{ij(h-1)}$:

$$d(\Delta y_{ijh};\Delta t_{ijh},\mu_{i})=\left\{\begin{array}{l} {\left(\frac{\Delta y_{ijh}}{\Delta t_{ijh}}-\mu_{i}\right)}^{2},r=0\\ 2\left[\frac{\Delta y_{ijh}}{\Delta t_{ijh}}\log\left(\frac{\Delta y_{ijh}}{\mu_{i}\Delta t_{ijh}}\right)-\left(\frac{\Delta y_{ijh}}{\Delta t_{ijh}}-\mu_{i}\right)\right],r=1\\ 2\left[\log\left(\frac{\mu_{i}\Delta t_{ijh}}{\Delta y_{ijh}}\right)+\frac{\Delta y_{ijh}}{\mu_{i}\Delta t_{ijh}}-1\right],r=2\\ 2\left\{\frac{{\left[\max\left(\Delta y_{ijh}/\Delta t_{ijh},0\right)\right]}^{2-r}}{\left(1-r)(2-r\right)}-\frac{\mu_{i}^{1-r}\Delta y_{ijh}}{(1-r)\Delta t_{ijh}}+\frac{\mu_{i}^{2-r}}{2-r}\right\},r\neq 0,1,2 \end{array}\right.$$

Define the RUL of the current time point $t_{i}$ as $R_{i}=\inf\left\{\gamma_{i},Y(t_{i}+\gamma_{i})\geq \omega |Y_{0\to i}\right\}$. The CDF and PDF of $R_{i}$ at the current time point $t_{i}$ are:

(28) $$F_{R_{i}}(\gamma_{i}|Y_{0\to i},\Theta )≅\Phi \left[\sqrt{\frac{m}{\mu }}\left(\mu \sqrt{\gamma_{i}}-\frac{\omega -y_{i}}{\sqrt{\gamma_{i}}}\right)\right]$$

and

(29) $$f_{R_{i}}(\gamma_{i}|Y_{0\to i},\Theta )=\frac{\mu \gamma_{i}+\omega -y_{i}}{2\gamma_{i}}\cdot \sqrt{\frac{m}{2\pi \mu \gamma_{i}}}\cdot \exp\left[-\frac{m}{2\mu }{\left(\mu \sqrt{\gamma_{i}}-\frac{\omega -y_{i}}{\sqrt{\gamma_{i}}}\right)}^{2}\right]$$

Thus, the statistical distribution of $R_{i}$ is determined by m and μ. Once the CDF and PDF of RUL are obtained, the RUL can be estimated using the monitoring data.

## 5. Case Study

Data from vibration signals of high-speed rail wheelset treads are collected and used to validate the proposed ME-based TEDP model, and comparisons are made against traditional models.

### 5.1. Data Collection

This study focuses on high-speed rail wheelset treads, tracking the degradation process of the wheels using vibration signals collected by a state monitoring system installed on the wheels. In this study, each train carriage is equipped with two power bogies, each containing four wheels [28]. A status monitoring system with four sensors and a processor is installed on the wheels. Over time, material defects and operational loads may cause small cracks beneath the wheel surface, eventually developing into surface fatigue cracks. When these defects interact with the railway, the vibration signals collected by the sensors can monitor the degradation process of the wheels.

**Figure 2:**
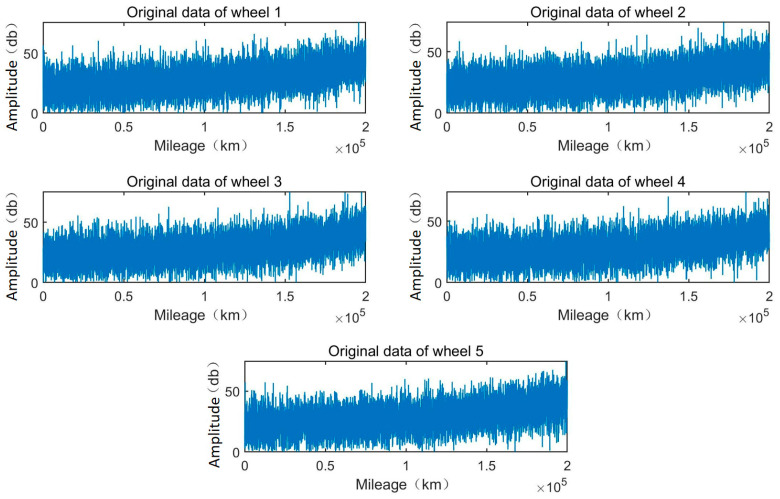
Original vibration signal of wheels 1 to 5.

This study presents the wheel degradation data of a high-speed train operating in China under real-world conditions. Vibration data from the train’s wheels were taken every 20 km over a total distance of 200,000 km. Data were obtained from five wheels on the same train. In total, 1000 data points were collected using high-precision vibration measurement equipment. The measurements were taken under various operational conditions, including different track types, weather conditions, and train speeds, ensuring that the data captured a wide range of real-world scenarios. The data collection process was carefully monitored to ensure accuracy and consistency, with regular calibration of the measurement devices and quality checks performed on the collected data. The data are shown in Figure 2.

### 5.2. Degradation Feature Extraction

For mechanical vibration signals, commonly used indicators of the mechanical state include time domain and frequency domain indicators. Given the sparse measurement frequency of the data, this study employs time domain features to represent the data state. Consequently, one or more degradation features are extracted from the raw signals collected over time, and the RUL is predicted based on the selected or fused features at a predetermined threshold. In time domain analysis, commonly used indicators and their typical formulas are shown in Table 2, with $s_{i}$ referring to the amplitude of the sampled data points and N representing the number of sampled data points for each sample. In this study, 12 features listed in Table 2 were extracted from the raw data.

**Figure 3:**
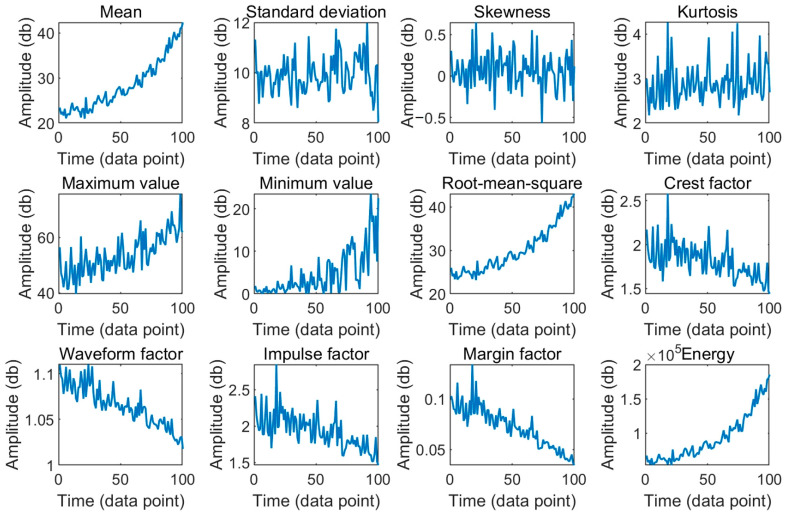
Feature extraction signal of wheel 1.

Time domain features are extracted from the datasets of all five wheels. Specifically, for each wheel, features are extracted from every 100 points of the 10,000 original data points in chronological order, yielding 100 feature data points. Using wheel 1 as an example, the time domain feature extraction results are shown in Figure 3. The extracted feature signals for the remaining four wheels are similar.

The data after time domain feature extraction still exhibit some volatility; however, certain features display more pronounced trends compared to others. Among all time domain features, the mean, root mean square, and energy features effectively reducing most random fluctuations and demonstrating a clear increasing trend. In practical scenarios, the degradation process of machinery is essentially irreversible; a faulty component, if not manually repaired, will not recover on its own. To reflect this irreversible degradation process, a suitable degradation characteristic should exhibit a monotonically increasing or decreasing trend, referred to as monotonicity [29]. Monotonicity is an intrinsic property of degradation characteristics, independent of their correlation with other factors, typically represented by a formula related to the sequence of degradation characteristics. This study employs an indicator proposed by Zhang et al. in 2014 to assess the monotonicity of degradation features [30] and selects the optimal feature among the mean, root mean square, and energy. The formula is as follows:

(30) $$Mon(X)=\frac{1}{K-1}\left|No.ofd/dx>0-No.ofd/dx<0\right|$$

Among them, $X={\left\{x_{k}\right\}}_{k=1:K}$ is the degenerate feature sequence, and $x_{k}$ represents the degenerate feature value at time $t_{k}$; K is the total number of degenerate eigenvalues in the sequence; $d/dx=x_{k+1}-x_{k}$ represents the difference between degraded feature sequences; No.ofd/dx>0 and represents the number of positive and negative differences, respectively. The values of Mon(X) range from 0 to 1, where higher scores indicate better the monotonicity performance.

The monotonicity index values of mean, root mean square, and energy are shown in Table 3.

Based on the indicator values presented in Table 2, there is no significant difference in the monotonicity of the mean, root mean square, and energy features. Among the data from the five wheels, the monotonicity indicator value of the mean feature is the largest among the three time domain features, particularly for wheel three, where it substantially exceeds the monotonicity values of the other two features. Consequently, this study selects the mean feature for subsequent parameter estimation and data processing.

To minimize random fluctuations in vibration signals and obtain a strictly monotonically increasing data sequence, this study uses order-preserving regression and exponential smoothing techniques. Sequential regression, commonly used in the medical field, derives response values that satisfy the order constraint relationship without altering the dosage order, resulting in a non-decreasing function [31].

After sequential regression, exponential smoothing is applied to ensure data monotonicity. This method calculates the weighted average of observed and predicted values from time t to forecast time t+1, with a single smoothing coefficient. As the time gap between observation and prediction increases, the weight of the observation value progressively decreases. The prediction formula is given as follows:

(31) $$F_{t+1}=\alpha Y_{t}+(1-\alpha )F_{t}$$

Here, $Y_{t}$ is the actual observation value of phase t; $F_{t}$ is the predicted value for the t period; α is the smoothing coefficient.

**Figure 4:**
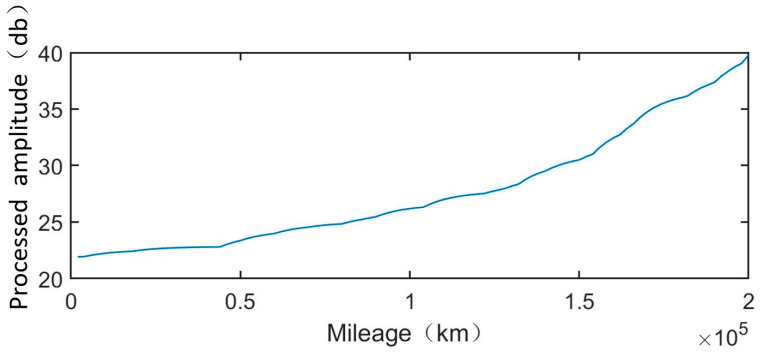
Vibration signal of wheels 1 to 5 after processing.

The processed amplitude data of wheel 1 is shown in Figure 4. The processed vibration signals for the remaining four wheels are similar.

### 5.3. Parameter Estimation

In this study, 100 data points were obtained for each wheel after feature extraction. To determine whether the degradation mechanisms differ across stages, each wheel’s 100 characteristic data points are divided into 10 degradation phases, equivalent to ten measurements under one stress value. The corresponding parameters are: $n=100,k=10,i=10,c_{i}=10$. Substitute the mean characteristic data of the five wheels into Equations (19) and (20) to derive the estimated values of the parameters, as shown in Table 4.

### 5.4. Mechanism Test

(1) Construction of MEF Samples

According to the estimation results for parameter $\hat{m}$ in Table 3, ${\hat{M}}_{1}-{\hat{M}}_{10}$ are constructed. For each degradation stage, i, ${\hat{M}}_{i}$ represents an MEF vector with five elements.

(2) Normality Test of MEF Estimation Values

SW tests are performed on each hypothesis testing sample ${\hat{M}}_{1}-{\hat{M}}_{10}$, all of which passed, indicating that the data groups follow a normal distribution and meet the conditions for subsequent testing.

(3) Mechanism Equivalence Testing of MEF Estimation Values

Testing showed that the degradation data of the ten stages of the train running gear tread passed the SW test, indicating that ${\hat{M}}_{i1},{\hat{M}}_{i2},{\hat{M}}_{i3},{\hat{M}}_{i4},{\hat{M}}_{i5}$ follows a normal distribution, i=1,2,⋯,10. Based on this, the study further compares the mean and variance of MEF estimates under different degradation phases using homogeneity of variance test and AVOVA to access the consistency of these normal distributions.

The Bartlett statistic is calculated for the data of the ten stages, and the test indicated that the variance of MEF estimation under the ten degradation stages can be considered equivalent.

$$Β=21.59<\chi_{0.01}^{2}(9)=21.667$$

After the variance equivalence are confirmed by Bartlett test, ANOVA is used to test whether the mean values of MEF across the 10 degradation stages are equivalent. The ANOVA result yielded a significance level of 0.789, which is significantly greater than 0.005, indicating that the null hypothesis of equivalence for the mean values of MEF across the ten degradation stages can be accepted.

Based on these results, it can be concluded that the failure mechanism of the tread remains consistent throughout the train’s operation from 0 to 200,000 km. Therefore, from the perspective of degradation mechanism equivalence, all degradation data collected under k=1−10 are selected as valid experimental data.

### 5.5. RUL Prediction

Based on the RUL prediction method described in Section 4.3, the estimation of RUL in traditional TEDP models is related to parameters λ, μ and r. For the Wiener process, the gamma process, and IG process, their parameters and RUL are calculated when the parameter r is fixed at 0, 2, and 3, respectively. As shown in Figure 4, when the mileage reaches $2\times 10^{5}$ km, the processed amplitude of the five wheels is approximately 40 dB, so the failure threshold is defined as 40 dB. Under this assumption, Table 5 presents the estimated RUL values obtained using the Wiener process, gamma process, IG process, traditional TEDP model, and ME-based TEDP model.

**Figure 5:**
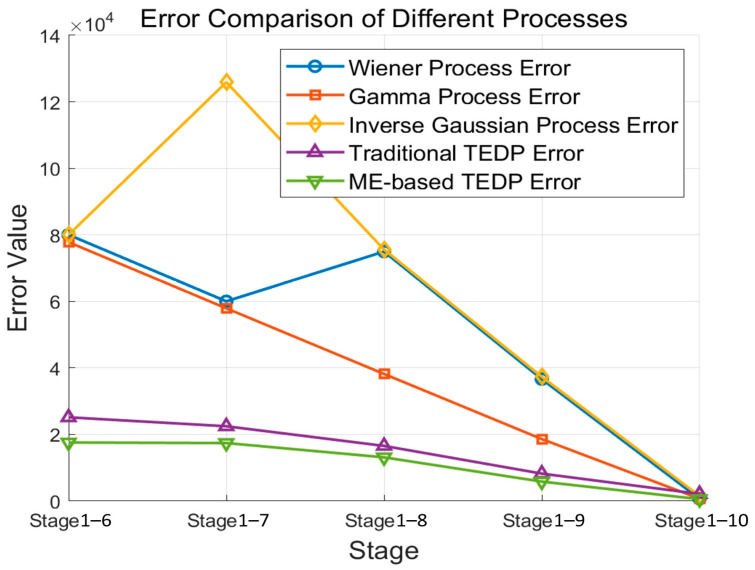
Comparison of errors among five models.

To enable a direct comparison of RUL estimation errors across models, Figure 5 shows the error values (estimated RUL minus actual RUL) for the five models as usage data increases.

Compared to the three standard degradation models—the Wiener process, gamma process, and IG process—the traditional TEDP model and ME-based TEDP model exhibit significantly better performance in predicting RUL. Notably, the ME-based TEDP model consistently achieves smaller prediction errors than the traditional TEDP model across all stages.

Additionally, this study compares the Akaike Information Criterion (AIC) and Bayesian Information Criterion (BIC) [32] for the Wiener, gamma, IG, traditional TEDP, and ME-based TEDP models using data from stages 1 through 10. Table 6 presents the results.

As shown in Table 6, the ME-based TEDP model achieves the best performance. This superiority among the five models is consistent with the conclusions drawn from the RUL prediction results. Furthermore, a comprehensive robustness analysis of the model parameters is performed, and the findings indicate that the ME-based TEDP model demonstrates substantial resilience to external perturbations, further validating its reliability in diverse operational scenarios.

## 6. Conclusions

This study focuses on the practical application of the TEDP model for mechanical component performance degradation modeling and lifetime prediction. Building on in-depth theoretical derivations and case analyses, this study provides a detailed assessment of the model’s applicability.

First, the study defines the TEDP model and applies the acceleration factor invariant principle to derive the equivalence conditions for degradation mechanisms within the TEDP framework. Based on this, the traditional TEDP model is refined into a more physically interpretable ME-based TEDP degradation model. This refinement introduces a novel approach that enhances the interpretability of degradation mechanisms, making it more aligned with real-world mechanical behaviors. Second, to address potential changes in degradation mechanisms, a multi-statistic method is employed to develop a process for testing the mechanisms of mechanical component performance degradation within the TEDP model. This multi-statistic approach enhances the robustness of the TEDP model by adapting to changes in degradation patterns, thereby ensuring more reliable predictions under varying operational conditions. Finally, the study introduces a parameter estimation and lifetime prediction method for the ME-based TEDP model. Engineering case studies demonstrate that the proposed mechanism enhances RUL prediction accuracy, significantly reducing prediction errors and confirming its broad applicability and accuracy. The comparison of absolute errors in RUL predictions, as shown in Figure 5, demonstrates that the proposed ME-based TEDP model and the associated lifetime prediction methodology significantly improve the accuracy and robustness of RUL predictions compared to traditional models, including the Wiener, gamma, IG, and conventional TEDP models.

Additionally, through error comparison analysis and model selection methods based on AIC and BIC criteria, this study evaluates the advantages and limitations of the TEDP model in mechanism equivalence analysis and product lifetime prediction. Although the model excels in many areas, it exhibits certain limitations, such as achieving comparable predictive performance to other degradation models in large sample scenarios. This finding highlights the need for future research aimed at enhancing the model’s adaptability and performance in diverse practical applications.

Future research directions include exploring more efficient data preprocessing techniques to improve model performance and accuracy, further developing model selection methods to identify optimal modeling approaches, and expanding TEDP applications to other product types or systems to enhance their effectiveness in modeling product failure mechanisms and lifetime prediction.

## Figures and Tables

**Table 1:** Special cases of ME-based TEDP model.

Model Parameter	Wiener Process Y(t)=ηt+σB(t)	Gamma Process $Y(t)\sim \Gamma (t;\beta_{1},\beta_{2})$	IG Process $Y(t)\sim IG(t;\delta_{1},\delta_{2})$
r	0	2	3
μ	η	$\beta_{1}/\beta_{2}$	$\delta_{1}$
m	$\eta /\sigma^{2}$	$\beta_{2}$	$\delta_{2}/\delta_{1}^{2}$
ω(q)	$q^{2}/2$	−ln(−q)	$-{\left(-2q\right)}^{1/2}$
κ(q)	q	−1/q	$-1/\left(2q^{2}\right)$

**Table 2:** Typical time domain feature calculation formulas.

Time Domain Feature	Equation
Mean	$\bar{s}=\frac{1}{N}\sum_{i=1}^{N}s_{i}$
Standard deviation	$\rho_{t}={\left(\frac{1}{N}\sum_{i=1}^{N}{\left(s_{i}-\bar{s}\right)}^{2}\right)}^{\frac{1}{2}}$
Root mean square	$RMS={\left(\frac{1}{N}\sum_{i=1}^{N}s_{i^{2}}\right)}^{\frac{1}{2}}$
Maximum	$s_{\max}$
Minimum	$s_{\min}$
Amplitude factor	$s_{\max}/RMS$
Waveform factor	$RMS/\left(\frac{1}{N}\sum_{i=1}^{N}\left|s_{i}\right|\right)$
Impulse factor	$s_{\max}/\left(\frac{1}{N}\sum_{i=1}^{N}\left|s_{i}\right|\right)$
Margin factor	$s_{\max}/{\left(\frac{1}{N}\sum_{i=1}^{N}\left|s_{i}\right|\right)}^{2}$
Energy	$\sum_{i=1}^{N}s_{i^{2}}$

**Table 3:** The monotonicity index values of mean, root mean square, and energy for different wheels.

	Time Domain Features	Monotonic Index Value
Wheel 1	Mean	0.12
	Root mean square	0.12
	Energy	0.12
Wheel 2	Mean	0.12
	Root mean square	0.10
	Energy	0.10
Wheel 3	Mean	0.16
	Root mean square	0.08
	Energy	0.08
Wheel 4	Mean	0.02
	Root mean square	0.02
	Energy	0.02
Wheel 5	Mean	0.12
	Root mean square	0.14
	Energy	0.14

**Table 4:** Parameter estimation results.

Degradation Stage k											
		1	2	3	4	5	6	7	8	9	10
Wheel 1	$\hat{\mu }$	0.037	0.053	0.086	0.127	0.147	0.102	0.220	0.243	0.370	0.422
	$\hat{r}$	3.282	2.453	3.002	2.453	1.351	1.678	2.062	2.028	0.574	1.302
	$\hat{\lambda }$	0.015	0.621	0.049	0.920	6.501	18.109	4.720	3.370	32.091	25.993
	$\hat{m}$	0.036	0.023	0.149	0.054	0.079	0.012	0.042	0.069	0.048	0.030
Wheel 2	$\hat{\mu }$	0.060	0.029	0.120	0.086	0.135	0.129	0.205	0.291	0.356	0.379
	$\hat{r}$	2.388	2.462	1.048	2.479	3.921	3.221	1.278	2.766	1.572	2.531
	$\hat{\lambda }$	1.202	0.458	16.021	1.437	0.315	0.242	13.844	1.754	9.735	3.727
	$\hat{m}$	0.017	0.012	0.056	0.018	0.009	0.044	0.046	0.064	0.057	0.061
Wheel 3	$\hat{\mu }$	0.031	0.081	0.071	0.120	0.100	0.189	0.191	0.273	0.318	0.486
	$\hat{r}$	1.745	3.255	2.203	1.228	1.172	0.192	2.925	4.139	1.882	2.498
	$\hat{\lambda }$	14.445	0.254	6.030	22.799	70.043	111.314	1.157	0.488	6.900	10.121
	$\hat{m}$	0.005	0.014	0.007	0.027	0.010	0.035	0.036	0.035	0.053	0.034
Wheel 4	$\hat{\mu }$	0.040	0.038	0.074	0.138	0.097	0.131	0.273	0.273	0.315	0.428
	$\hat{r}$	3.328	1.552	0.922	2.002	2.794	2.467	2.431	1.060	1.636	1.204
	$\hat{m}$	0.034	32.087	51.546	3.753	0.396	1.223	10.300	36.591	7.595	9.834
	$\hat{m}$	0.016	0.005	0.024	0.037	0.038	0.042	0.015	0.025	0.063	0.085
Wheel 5	$\hat{\mu }$	0.064	0.090	0.084	0.088	0.122	0.099	0.239	0.304	0.277	0.453
	$\hat{r}$	2.493	2.014	3.031	2.251	2.445	2.473	0.830	8.510	1.227	1.508
	$\hat{\lambda }$	2.497	1.674	1.374	0.783	0.806	1.258	94.538	0.012	20.845	14.586
	$\hat{m}$	0.007	0.052	0.005	0.061	0.059	0.026	0.013	0.011	0.036	0.046

**Table 5:** Expected lifetime results.

**Stage**	**RUL Expectancy (km)**	**Wiener Process (km)**	**Gamma Process (km)**	**Inverse Gaussian Process (km)**	**Traditional TEDP (km)**	**ME-Based TEDP (km)**
1–6	8 × 10^4^	8.33 × 10^−14^	2.21 × 10^3^	4.43 × 10^−17^	5.48 × 10^4^	6.24 × 10^4^
1–7	6 × 10^4^	1.48 × 10^−16^	2.10 × 10^3^	1.86 × 10^5^	3.75 × 10^4^	4.26 × 10^4^
1–8	4 × 10^4^	1.15 × 10^5^	1.82 × 10^3^	1.16 × 10^5^	2.34 × 10^4^	2.69 × 10^4^
1–9	2 × 10^4^	5.66 × 10^4^	1.38 × 10^3^	5.73 × 10^4^	1.18 × 10^4^	1.42 × 10^4^
1–10	0	6.80 × 10^2^	7.07 × 10^2^	1.52 × 10^3^	2.13 × 10^3^	5.62 × 10^2^

**Table 6:** AIC and BIC of each model.

	Wiener Process	Gamma Process	Inverse Gaussian Process	Traditional TEDP	ME-Based TEDP
AIC	−463.4044	−719.9949	−648.7202	−1101.5236	−1323.8538
BIC	−454.9952	−711.5858	−640.3111	−1088.9099	−1311.2401

## Data Availability

Datasets are available upon request to the corresponding author.
